# Wilms' Tumor Protein 1 and Enzymatic Oxidation of 5-Methylcytosine in Brain Tumors: Potential Perspectives

**DOI:** 10.3389/fcell.2018.00026

**Published:** 2018-03-22

**Authors:** Ashley Ramsawhook, Alexey Ruzov, Beth Coyle

**Affiliations:** ^1^Wolfson Centre for Stem Cells, Tissue Engineering and Modelling (STEM), Centre for Biomolecular Sciences, University of Nottingham, Nottingham, United Kingdom; ^2^Children's Brain Tumour Research Centre, Medical School, Queen's Medical Centre, University of Nottingham, Nottingham, United Kingdom

**Keywords:** Wilms' Tumor 1, DNA demethylation, epigenetics, brain tumors, glioblastoma, ependymoma, medullobastoma, TET proteins

## Abstract

The patterns of 5-methylcytosine (5mC) and its oxidized derivatives, 5-hydroxymethylcytosine, 5-formylcytosine, and 5-carboxylcytosine (5caC) are reportedly altered in a range of cancers. Likewise, Wilms' Tumor protein 1 (WT1), a transcription factor essential for urogenital, epicardium, and kidney development exhibits aberrant expression in multiple tumors. Interestingly, WT1 directly interacts with TET proteins that catalyze the enzymatic oxidation of 5mC and exhibits high affinity for 5caC-containing DNA substrates *in vitro*. Here we review recent developments in the fields of Tet-dependent 5mC oxidation and WT1 biology and explore potential perspectives for studying the interplay between TETs and WT1 in brain tumors.

## Developmental basis for aberrant expression of wilms' tumor protein 1 in cancers

Wilms' Tumor protein 1 (WT1) is a Kruppel-like transcription factor important for development of the mammalian embryonic kidney, urogenital system and epicardium (Roberts, 2005) that exhibits developmental and tissue specific expression patterns in mammals (Dressler, 2009; Hastie, 2017). Inactivating mutations occurring in *WT1* during embryonic kidney development result in the formation of the pediatric renal neoplasm Wilms' tumor (WT) (Pritchard-Jones et al., 1990).

Onset of WT1 expression in murine embryos commences at 9 days post coitum (dpc) where the protein is detectable in the parietal epithelium lining the coelomic cavity (Armstrong et al., 1993). At this stage *WT1* mRNA transcripts (Pritchard-Jones et al., 1990) localize to the intermediate mesenchyme/mesoderm (Pelletier et al., 1991b; Vize et al., 1997). At 11 dpc, WT1 expression is detected in the metanephric mesenchyme, presumptive spinal cord motor neurons, gonads, and ureter (Armstrong et al., 1993). Significant increase in WT1 expression is observed in the metanephric mesenchyme and nephrogenic condensates at 12.5 dpc, whereas gonadal WT1 expression remains level at this stage (Armstrong et al., 1993). Between 13.5 and 15 dpc, developing epicardium, podocytes of the glomerulus, endothelial, and stromal cells of the ovaries and uterus, Sertoli cells of the testis, retinal ganglia of the eye and ependymal cells of the fourth ventricle in the brain all possess positive immunostaining for WT1 (Sharma et al., 1992). At 20 dpc, WT1 is strongly expressed in kidney glomeruli and is weakly present in the eye and tongue (Mundlos et al., 1993). These periods of murine embryonic development correlate to 28–70 dpc in human embryos (Reidy and Rosenblum, 2009). In murine and human adult tissues, WT1 is present in multiple genitourinary structures, haematopoietic stem cells, kidney glomeruli, and podocytes, ependymal cells of the spinal cord and the *area postrema* of the medulla (Huang et al., 1990; Mundlos et al., 1993; Ramani and Cowell, 1996; Clark, 2006; Nakatsuka et al., 2006).

Homozygous deletion of *WT1* (*wt1*^−/−^) in mice of C57BL/6 genetic background is embryonically lethal mainly due to genitourinary, kidney, and cardiac complications occurring between 12 and 15 dpc (Herzer et al., 1999). Specifically, *wt1*^−/-^ mice exhibit lack of seminiferous tubules, complete lack of spleen and uretic bud formation in kidneys and failure of epicardium to undergo epithelial to mesenchymal transition (EMT) (Herzer et al., 1999). In a developmental context, during embryonic kidney formation, *WT1* functions as a proto-oncogene, inducing proliferation of metanephric mesenchyme pluripotent progenitor cells required to respond to inductive WNT9b signals from the invading uretic bud to undergo mesenchymal to epithelial transitioning (MET) into glomerular podocytes (Hohenstein and Hastie, 2006). Metanephric mesenchyme cells originate from intermediate mesoderm arising at the gradient boundary of inductive bone morphogenetic protein (BMP) signals from the Splanchnic/lateral plate mesoderm and repressive retinoic acid (RA) signaling from the sixth somite of the paraxial mesoderm (Dressler, 2009).

WT1 exhibits a biphasic (at 10 and 12 dpc) pattern of expression in the developing kidney, specifically in (1) intermediate mesenchymal stem cells and mesonephric progenitors prior to their epithelial differentiation, and (2) during differentiation of epithelia in cap mesenchyme to glomerular podocytes, S-shaped bodies, comma shaped bodies, and renal vesicles at 12 dpc (Armstrong et al., 1993; Wilm et al., 2005). At 10 dpc, WT1 is required for the differentiation of mesonephric mesenchyme progenitors into transiently existing caudal tubular structures which function as a primitive temporary kidney (Wilm et al., 2005). In concordance with studies revealing that mesonephric progenitor induction at 10 dpc is regulated by the WT1 signaling-related genes including *Pax2* and *Six2* (Dressler et al., 1990; Dressler and Douglass, 1992), WT1 ablation in these progenitors results in significant reduction of caudal mesonephric tubules (Kreidberg et al., 1993). At 12 dpc, *wt1*^−/−^ murine embryos fail to induce uretic bud formation and hence patterning of the metanephric mesenchyme and subsequent glomerular structures cannot develop, culminating in embryonic lethality (Kreidberg et al., 1993). In both instances of WT1-contingent development (i.e., mesonephric and metanephric mesenchyme differentiation), WT1 is required to regulate the mesenchymal to epithelial transition of mesodermally derived blastemal progenitors toward nephrogenic capillary epithelium (Sainio et al., 1997). In the developing human fetus circa 11–12 weeks of gestation, WT1 protein expression has been observed in kidney glomerular podocytes, Bowman's Capsule parietal epithelium, mesothelial cells covering surfaces of ovaries and testis, somatic skeletal muscle, the tongue, and radial glia of the spinal cord (Parenti et al., 2013; Magro et al., 2015). WT1 expression in glomerular podocytes is maintained throughout adult life which may implicate a role in metanephric mesenchyme maintenance of stemness and organ repair (Guo et al., 2002).

Approximately 5–20% of Wilms' Tumors cases exhibit somatic *WT1* gene mutations which tend to be sporadic bi-allelic aberrations occurring in blastemal progenitors (Kaneko et al., 2015). The inherited nature of mono-allelic germline *WT1* mutations or deletions occurring within chromosomal regions 11p13 predisposes individuals to Wilms' Tumor formation and account for ~5% of WT cases (Ruteshouser and Huff, 2004; Charlton et al., 2017). *WT1* null mutants in mesonephros pluripotent progenitors of the intermediate mesoderm exhibit morphology and genotypic characteristics of paraxial mesoderm-derived mesenchymal stem cells i.e., they possess adipogenic, chondrogenic, and osteogenic lineage differentiation potential (Royer-Pokora et al., 2010). Histologically, Wilms' Tumors exhibit a highly disorganized heterogeneous cell population with blastemal, stromal, undifferentiated mesenchymal, and epithelial cells represented (Grosfeld, 1999; Scott et al., 2006). Failure of nephrogenic mesenchyme progenitors to differentiate into pretubular aggregates, renal vesicles, and eventually glomerula podocytes is attributable to *WT1* mutations (Morizane et al., 2015). However, mutation of early nephrogenic progenitor specific genes such as *Cited1* and *Six2* results in stabilization and accumulation of β-catenin, thus inducing oncogenic targets of *WNT* signaling (Beckwith et al., 1990; Rivera and Haber, 2005; Huang et al., 2016). Mutations in *WT1, Cited1*, and *Six2* result in Wilms' Tumors when they occur in pluripotent nephrogenic progenitors but not in stromal progenitors (Charlton et al., 2017). Moreover, Wilms' tumors exhibit elevated expression of genes pertaining to early kidney development such as those involved in uretic bud induction and nephrogenic mesenchyme patterning (e.g., *Pax2, Pax8, Gata3, GDNF*, and *Wnts 4* and *9b*) and down-regulation of late development genes (Reidy and Rosenblum, 2009; Morizane et al., 2015).

Nephrogenic rests (precursors to malignant Wilms' tumors) formed as a consequence of mono-allelic inactivation transformed into the eponymous renal malignancy upon secondary *WT1* allele inactivation, a classic example of Knudson's two hit hypothesis (Ruteshouser et al., 2008; Royer-Pokora et al., 2010; Kaneko et al., 2015). The nephrogenic rests themselves occur more frequently as a consequence of somatic mono-allelic *WT1* mutation in sporadic Wilms' Tumors (90–95%) compared to familial Wilms' tumor germline *WT1* mutations (1–2%; Cardoso et al., 2013). Nephrogenic rests consist of primitive undifferentiated embryonic blastemal cells which are observed predominately in intralobar nephrogenic rests (ILNR) whereas undifferentiated mesenchymal stromal progenitors populate perilobar nephrogenic rests (PLNR) (Royer-Pokora et al., 2010; Charlton et al., 2017). *WT1* mutations are associated with ILNR which persist in early kidney development (10 dpc) and is commensurate with induction of WT1 expression in pluripotent mesonephros during this time (Wilm and Muñoz-Chapuli, 2016). The absence of WT1 mutations in PLNR may be due to a WT1-independent and restricted lineage differentiation potential of metanephric stromal progenitors (Wilm and Muñoz-Chapuli, 2016). Collectively, this is suggestive of a developmentally early WT1-dependent paradigm of patterning and differentiation of the intermediate mesoderm and metanephric mesenchyme (Pelletier et al., 1991a; Royer-Pokora et al., 2010). However, the increased cellular proliferation rate resulting in the formation of neoplasms with *WT1* mutant genotype evident in 5–20% of Wilms' Tumor cases implicates a tumor suppressor role for WT1, indicating both proto-oncogene and tumor suppressor functions for this transcription factor (Algar et al., 1996; Yamagami et al., 1996; Inoue et al., 1998; Menke and Van Der Eb, 1998; Tsuboi et al., 1999; Loeb and Sukumar, 2002; Li et al., 2003; Tatsumi et al., 2008).

## Impact of ectopic WT1 expression on tumorigenesis

*WT1* wildtype mRNA has been detected in a range of malignancies including oesophageal, gastric, colorectal, pancreatic, biliary, lung, prostate, renal, breast, cervical, ovarian, endometrial, soft tissue, and brain cancers, indicative of a possible oncogenic contribution of ectopic *WT1* expression in tumorigenesis (Nakatsuka et al., 2006). Studies performed on mouse lung cancer cells revealed a WT1-dependent mechanism of oncogenic KRAS induced proliferation (Vicent et al., 2010). Human and mouse lung cancer cells with ablated *WT1* reduced proliferation and triggered senescence, highlighting anti-apoptotic consequences of WT1 ectopic overexpression (Vicent et al., 2010). These results have been corroborated by immunohistochemistry by both N- and C-terminus-specific WT1 antibodies (Nakatsuka et al., 2006). Interestingly, although WT1 is not expressed in astrocytes and is found only in brain endothelium in healthy adult humans (Bourne et al., 2010), its elevated expression is common for brain tumors such as pilocytic astrocytoma (grade I), anaplastic astrocytoma (grade III), and glioblastoma multiforme (grade IV) (Nakahara et al., 2004; Izumoto et al., 2008; Chiba et al., 2010; Rauscher et al., 2014). Increasing *WT1* levels are commensurate with increasing tumor grade and associated with poor patient prognosis (Nakahara et al., 2004; Izumoto et al., 2008; Chiba et al., 2010; Rauscher et al., 2014). Increased incidence of high *WT1* expression levels have been observed to correlate with severity of tumor grade of pediatric ependymoma with highest levels present in grade III anaplastic ependymoma (Yeung et al., 2013). *WT1* overexpression was observed in 98% of glioblastoma primary cell samples and 83% of anaplastic astrocytomas compared to 53% of grade II oligodendroglioma and pilocytic astrocytomas (Schittenhelm et al., 2008). Short hairpin RNA (shRNA) molecules targeting *WT1* mRNA transiently silence *WT1* gene expression and reduce glioblastoma cell proliferation, viability, and invasion ability suggesting an oncogenic role for WT1 in these malignancies as opposed to the tumor suppressor role this protein plays in Wilms' tumor (Schittenhelm et al., 2008; Clark et al., 2010; Kijima et al., 2016).

Closer inspection of the WT1 structure provides clues to its functional role in tumorigenesis. WT1 is encoded by the corresponding gene located on chromosome 11p13 that is composed of 10 exons with exons 5 and 9 undergoing alternate splicing to form functionally different isoforms (Call et al., 1990). The WT1 gene harbors two ATG start codons, with one at the conventional +1 site and the second residing between exons 1 and 2, translation from which, synthesizes a curtailed protein variant at the N terminal (Dallosso et al., 2004). Full length WT1 protein consists of an N terminal RNA recognition motif (RRM), dimerization domain, activation domain, alternatively spliced 17 amino acid auxiliary interaction domain encoded by exon 5 and 4 and C-terminal Cysteine-Histidine (C2-H2) zinc finger domains (Bickmore et al., 1992). Zinc fingers 1–4 are encoded by exons 7, 8, 9, and 10, respectively (Ladomery and Dellaire, 2002). Alternate splicing between zinc fingers 3 and 4 generates two isoforms either possessing or absent for lysine-threonine-serine (KTS^+/−−^) amino acid triplet sequence located between zinc fingers 3 and 4 (Clark et al., 2007). WT1 Zinc fingers 1 & 2 can interact with p53 facilitating its sequestration and stabilization for subsequent ubiquitination and proteolysis (Maheswaran et al., 1995). Stabilization of p53 by WT1 prevents induction of pro-apoptotic pathways (Maheswaran et al., 1995). This occurs in a temporally and spatially regulated fashion during embryonic kidney development as nephrogenic progenitor cells (NPCs) within the metanephric mesenchyme are permitted to undergo differentiation to pretubular aggregates, renal vesicles and eventually glomerula podocytes at E8.0 (Kreidberg et al., 1993; Dressler, 2009; Brown et al., 2011; Short et al., 2014; Kann et al., 2015). Full length WT1 DNA binding domain is required for its physical interaction with p53 and as such, only +KTS and not –KTS isoforms can bind to wildtype p53 to stabilize the protein (Maheswaran et al., 1995). Surprisingly, studies in osteosarcoma models indicated that the pro-apoptotic effect of p53 expression can be inhibited by WT1 +KTS isoforms but not –KTS isoforms (Maheswaran et al., 1995; Mayo et al., 1999; Loeb, 2006). One explanation for this observation may include the WT1 facilitated inhibition of E6/E6AP mediated p53 ubiquitination and consequent proteolysis. Upon complexing with p53, WT1, may sterically prevent proteolytic degradation of p53 by occluding its ubiquitination sites from targeting by E6 ubiquitin ligase (Yamanouchi et al., 2014). In a murine model of ovarian cancer, transient overexpression of WT1 −17aa/-KTS isoform was linked to significant increases in cellular proliferation, migration, and angiogenesis leading to a significant reduction of mouse survival time (Yamanouchi et al., 2014). These results were in stark contrast to over-expression of +KTS isoforms which did not significantly affect these parameters and the +17aa/–KTS splice variant which induced apoptosis via suppression of *EGFR* transcription (Yamanouchi et al., 2014).

## Reconfiguration of the DNA methylome in brain tumors

DNA methylation, denoted by 5-methylcytosine (5mC) occurring on the 5′ carbon of cytosine within a CpG dinucleotide has been well characterized as a transcriptionally repressive mark (Bird, 2002). Owing to its mutagenic potential to deaminate to thymine, 5mC is present on 70% of CpG cytosines, accounting for ~1% of mammalian genomes (Youssoufian et al., 1986). Methylation of CpG cytosines is catalyzed by maintenance and *de novo* DNA methyltransferase (DNMT) enzymes DNMT1 and DNMT3A/B, respectively (Kim et al., 2002). Genome wide methylation studies have compared 5mC distribution among DNA sequences of varying CpG density including low CpG density, intermediate CpG density, high CpG density, differentially methylated regions (DMRs), and long terminal repeats (LTRs) (Weber et al., 2007). Reduced representation bisulphite sequencing (RRBS) analysis of these regions revealed an inversely proportional relationship between CpG density and methylation level in sequenced fragments of 40–220 base pairs (bp) across 21 million reads (Weber et al., 2007). In contrast, methylation levels were significantly higher among low density CpG regions which correlated with the presence of transcriptionally permissive or active histone mark histone 3, lysine 4 trimethylation (H3K4me3), and dimethylated lysine 4 tails on histone 3 (H3K4me2) (Weber et al., 2007). Amongst the minute fraction of methylated CpGs (0.3%) in the vastly unmethylated high density CpG island sequences, occurrence of CpG methylation correlated with transcriptionally repressive histone mark H3K27me3 (Weber et al., 2007).

High-performance liquid chromatography (HPLC) analysis of 5mC distribution between different tissues detected highest levels of this modification in the brain (Kriaucionis and Heintz, 2009). Isotope labeled liquid chromatography-coupled mass spectrometry studies investigating 5mC levels in the adult human brain depicted significant differences in the distribution of this epigenetic mark between different brain regions (Kraus et al., 2012). Frontal and occipital lobes of the cerebral cortex scored highest for 5mC levels whilst frontal and occipital white matter tracts exhibited significantly lower 5mC presence (Kraus et al., 2012). Clues as to the nature of this observed disparity in 5mC yield between different organs and even distinct regions of the same tissue may be attributable to the relatively recent discovery of oxidized forms of 5mC and the mechanism governing their generation (Kriaucionis and Heintz, 2009; Khare et al., 2012).

The Ten-Eleven Translocase (TET) proteins, homologous to J-Base binding proteins (JBP) discovered in *Trypanosome bruceii*, can recognize and oxidize 5mC to 5-hydroxymethylcytosine (5hmC), 5-formylcytosine (5fC), and 5-carboxylcytosine (5caC) (Tahiliani et al., 2009; Ito et al., 2011). According to a growing body of experimental evidence, these oxidized forms of 5mC (referred together as oxi-mCs) may play specific roles in epigenetic regulation of gene expression (Wu and Zhang, 2010; Seisenberger et al., 2012; Hackett et al., 2013; Smith and Meissner, 2013; Guo et al., 2014; Hu et al., 2014; Lewis et al., 2017). Importantly, both HPLC and the restriction endonuclease enzyme-facilitated oligonucleotide probe array hybridization mapping at single base resolution data demonstrate the relative enrichment of 5hmC within healthy human brains relative to other organs such as liver, kidney, pancreas, and heart (Khare et al., 2012).

In a recent study, Brain tumors representing all World Health Organization (WHO) classifications (grades I-IV) ranging from grade I temporal lobe pilocytic astrocytoma and grade II cervical spine ependymoma to grade III cerebellum anaplastic astrocytoma and grade IV parietal lobe glioblastoma multiforme were interrogated for the presence and magnitude of 5mC and 5hmC marks (Kraus et al., 2012). The WHO tumor grades increase with disease severity as characterized by tumor cell proliferative index, nuclear abnormality, necrosis, micro-vascularization, invasiveness, and anti-correlate with patient prognosis and survival rate (Louis et al., 2007). In these experiments examining the quantity and distribution of 5mC and 5hmC between healthy brain and brain tumor specimens, mass spectrometry-validated immunohistochemical analysis revealed no significant difference in 5mC levels between healthy brains and tissue matched tumors of varying grades, isolated from multiple locations (Kraus et al., 2012). Contrasting with this, the same study revealed heterogeneous 5hmC signatures with greater proportion of positive staining in lower (grade I) tumors (16.7%) relative to higher (grade IV) ones (1.43%) in the analyzed brain tumors. Highest 5hmC signal intensity (5hmC/dG ratio) captured via mass spectrometry was observed also in grade I tumors (0.22%) whereas grade IV tumors exhibited the lowest 5hmC signal intensity (0.078%). Frontal and occipital cortex regions and even their lower white matter tracts scored considerably higher 5hmC levels relative to all brain tumors analyzed (Kraus et al., 2012).

Bisulphite and oxidative bisulphite sequencing employing sodium bisulphite and potassium perruthenate chemistries, respectively, facilitate the discrimination of 5mC from unmodified cytosine and 5fC from 5hmC (Johnson et al., 2016). Sequencing of methylated and demethylation intermediate attached bases is possible at single base resolution and has consequently revealed the overall depletion of 5hmC from glioblastoma cells (Johnson et al., 2016). Examination of 30 glioblastoma cell lines and primary tissues exposed distinct 5hmC occupancy at CpG oceans and shores which are comprised of low density CpG frequency relative to CpG islands and shores and, thus, possess intermediate levels of 5mC enrichment. This highlights a DNA demethylation intermediate role for 5hmC, as 5mC erasure from sequences augments transcriptional status of genes under regulation (Johnson et al., 2016). The CpG shelves which displayed highest 5hmC enrichment (75%) localized within a window of ~5 kilobases (Kb) upstream from transcriptional start sites coinciding with enhancer and super-enhancer territory (Johnson et al., 2016). These genomic features characterized by association with active chromatin elements such as H3K27 acetylation (H3K27Ac), positively correlated with transcription of pathogenicity related genes in the aggressive glioblastoma cell line U87 (Johnson et al., 2016). In concurrence with previously mentioned studies highlighting the correlation of depletion of 5hmC levels with the tumor grade increase (Orr et al., 2012), Recursive Partition Mixture Model (RPMM) clustering analysis of 5hmC occupancy in glioblastoma cells linked the cytosine modification to prolonged patient survival, an index associated with pro-neural subtypes of glioblastoma (Verhaak et al., 2010; Johnson et al., 2016). Despite 5hmC depletion within aggressive brain tumor grades, its presence within genomic features appears critical to glioblastoma pathogenicity with the enrichment of 5hmC identified in gene regulatory regions upstream of core glioblastoma signaling genes such as epidermal growth factor receptor vIII (EGFRvIII) and cyclin-dependent kinase 6 (CDK6) (Johnson et al., 2016).

## WT1 may modulate epigenetic signatures in tumors

Whilst *WT1* over-expression and null mutations have both been documented in cancer, implicating its dual properties as a proto-oncogene and tumor suppressor (Algar et al., 1996; Yamagami et al., 1996; Inoue et al., 1998; Menke and Van Der Eb, 1998; Tsuboi et al., 1999; Loeb and Sukumar, 2002; Li et al., 2003; Tatsumi et al., 2008), recent evidence has begun to demystify potential epigenetic regulatory functions of this protein in malignant neoplasms (Akpa et al., 2015). Thus, according to several studies, WT1 is able to prime metanephric mesenchyme progenitors for differentiation triggered by inductive WNT9b signals from the uretic bud via the repression of Enhancer of Zeste Homologue 2 (*EZH2*) transcription (Akpa et al., 2015). The consequent reduction of trimethylated lysine 27 on histone 3 (H3K27me3) enables transcription of beta-catenin (*CTNNB1*) in these progenitors and their subsequent nephrogenic differentiation (Akpa et al., 2015). In *Wt1*^+/−^ mutant cells, progenitors cannot respond to WNT9b induction and thus proliferate into nephrogenic rests that transform into malignant tumors upon *wt1* ablation (Dressler, 2009; Akpa et al., 2015).

**Figure 1:**
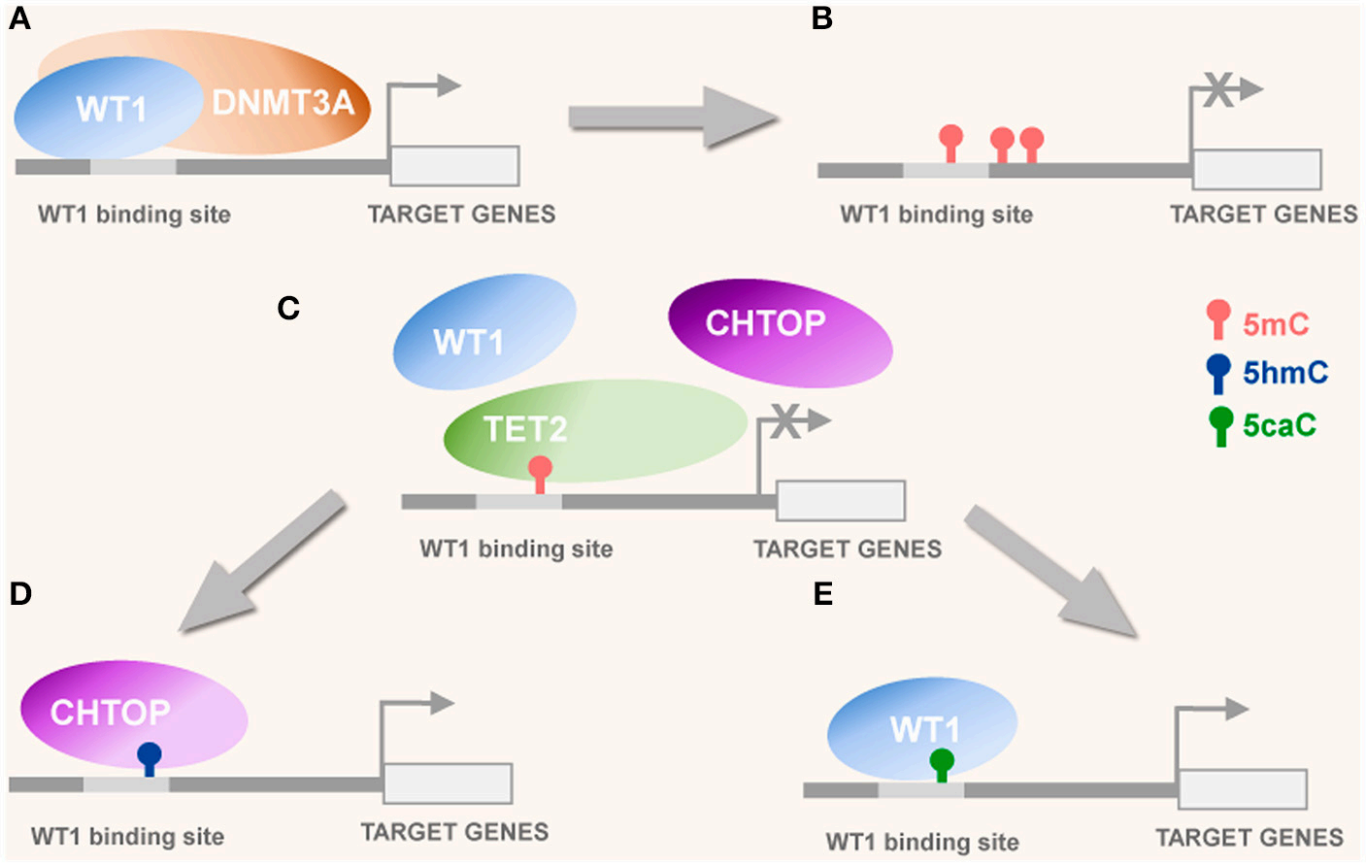
Potential interplay of WT1 with DNMT3A and CHTOP. **(A)** WT1 recruits DNMT3A to unmethylated CpGs or nearby WT1 binding sequences (Szemes et al., 2012) leading to *de novo* methylation of unmethylated cytosines by DNMT3A and consequently, transcriptional repression of WT1 target genes **(B)**. **(C)** Tet protein mediated oxidation of 5 mC to oxi-mC derivatives attracts oxi-mC reader proteins WT1 and CHTOP competing for the same DNA binding sequence. **(D)** 5mC oxidation to 5hmC attracts CHTOP to WT1 binding site leading to histone demethylation and transcriptional activation. **(E)** 5mC oxidation to 5caC, attracting WT1, which possesses high 5caC binding affinity may also result in transcriptional activation of its target genes.

Aberrant DNA methylation has been well documented in cancers where both hypermethylation of tumor suppressor genes and hypomethylation of retrotransposons, IAPs and proto-oncogenes are considered *bona fide* tumorigenic conditions (Ehrlich, 2002; Baylin, 2005; Baylin and Ohm, 2006; Hinoue et al., 2012). In addition, as oxi-mCs may serve as intermediates in the DNA demethylation process (Jones and Liang, 2009; Tahiliani et al., 2009; He et al., 2011; Shen et al., 2013; Dawlaty et al., 2014) these modifications may also be functionally involved in tumorigenesis (Dawson and Kouzarides, 2012; Ehrlich and Lacey, 2013; Eleftheriou et al., 2015; Tian et al., 2016; Ramsawhook et al., 2017). Correspondingly, TET proteins may display tumor suppressor activity and their mutations, generating defective or non-functional proteins may result in tumorigenesis (Ko et al., 2010). Thus, TET2 protein has been implicated in the development of hematological malignancies such as acute myeloid leukemia (AML), chronic myelomonocytic leukemia (CMML), and Myelodysplastic syndrome (MDS) (Tefferi et al., 2009). Interestingly, WT1 has been demonstrated to directly recruit DNMT3A to unmodified CpG cytosines (Figure 1) within gene regulatory sequences (Szemes et al., 2012). Moreover, WT1 has been demonstrated to bind directly to TET2 and TET3 recruiting them to their potential target sequences in acute myeloid leukemia models where mutation of WT1 resulting in its inactivation is accompanied with significant locus specific diminishing of 5hmC levels, a phenotype mimicked by mutations in the isocitrate dehydrogenase genes (*IDH1/2*) (Verhaak et al., 2010; Rampal et al., 2014; Kelly et al., 2017). The *IDH1* mutations have been linked to aberrant DNA methylation signatures in AML (Rampal et al., 2014) and secondary pro-neural glioblastoma (Verhaak et al., 2010; Turcan et al., 2012; Rampal et al., 2014; Kelly et al., 2017). In AML and pro-neural glioblastoma, a CpG island methylator phenotype (G-CIMP) is common (Noushmehr et al., 2010; Turcan et al., 2012) and hypermethylation of non-housekeeping genes has been observed (Sturm et al., 2012; Hughes et al., 2013; Weisenberger, 2014). Substitution mutations such as arginine 132 replaced by histidine (R132H) mutate *IDH1*, erroneously converting isocitrate to 2-hydroxyglutarate instead of alpha-ketoglutarate, the canonical co-factor required by TET enzymes for oxidation of 5mC (Rampal et al., 2014). The consequent inhibition of TET enzyme activity correlates with significant 5mC hypermethylation and reduction in 5hmC observed in AML cells (Rampal et al., 2014). Similar effects of hypomethylation and 5hmC reduction are characteristic of *Tet2* missense mutations that either result in its attenuated or abrogated catalytic ability (Konstandin et al., 2011). Interestingly, the aberrant methylation signatures observed in AML can be recapitulated in cells with wildtype *IDH1*&*2* and *Tet2* but mutant *WT1* (Rampal et al., 2014). Correspondingly, a negative correlation of *IDH1&2* and *Tet2* mutations with mutations of *WT1* was reported for a cohort of 398 AML patients (Rampal et al., 2014). Moreover, a significant reduction in 5hmC levels was demonstrated for these WT1 mutants compared to IDH1&2, TET2, and WT1 wildtype AML background controls (Rampal et al., 2014). Furthermore, next generation sequencing-based analysis revealed the existence of differentially methylated and hydroxymethylated regions between the wildtype AML controls and *WT1* mutants, with the majority of 5hmC peaks localizing to enhancers and distal regulatory regions whereas differential 5mC peaks between wildtype and mutants clustered around transcriptional start sites (Rampal et al., 2014). The 5hmC levels at differentially hydroxymethylated distal enhancers were lower in *WT1* AML mutants compared to controls and differentially methylated regions at transcriptional start sites were more greatly enriched for 5mC in *WT1* mutants (Rampal et al., 2014). This may suggest a transcriptional priming role for 5hmC in haematopoiesis which when perturbed by *WT1* mutations, may contribute to tumorigenesis (Ko et al., 2010; Yang et al., 2013). In glioblastoma multiforme, WT1 over-expression may facilitate competition or co-operative binding with other transcription factors capable of recognizing 5hmC occupancy at promoter and enhancer sequences (Takai et al., 2014). Intriguingly, DNA immunoprecipitation and subsequent sequencing (DIPSeq) demonstrate possible interactions between cytosine modifications and protein complexes capable of “reading” them and altering transcriptional status of associated genes (Takai et al., 2014). Core GBM signaling pathway genes e.g., *BRAF, AKT, EGFR*, & *CDK6* possessed 5hmC enrichment at their promoters and intergenic regions which positively correlated with chromatin target of PRMT1 (CHTOP) promoter presence and binding and consequent transcriptional activation of these genes (Takai et al., 2014). Excitingly, WT1 and CHTOP share binding affinity for the same promoter sequence (Figure 1), implicating WT1 as a strong contender for influencing sustained pathogenic transcriptional signaling in glioblastoma (Hashimoto et al., 2015).

In addition to its interaction with TET proteins, recent studies suggest that WT1 can also specifically bind certain oxi-mCs (Hashimoto et al., 2014, 2016). WT1, a member of the Early Growth Response family (EGR) of transcription factors shares DNA binding consensus sequence 5′-GCG(T/G)GGGCG-3′ (EGR-1 consensus) with its fellow family members EGR1 and Zif268 (Stoll et al., 2007). WT1 possesses oxi-mC vs. mC discrimination capabilities in contrast to 5mC vs. unmodified cytosine recognition as demonstrated by EGR1 and Zif268 (Hashimoto et al., 2014). Amongst oxi-mC derivatives, WT1 displays preferential binding affinity for 5caC over 5hmC and 5fC. Additionally, preferential binding affinity for 5caC is enhanced by presence of asymmetric methylation on the same transcription factor “recognition strand” and by occupancy of 5mC at CpG sites on the complementary strand (Hashimoto et al., 2016). This is indicative of specific epigenetic configurations on DNA sequences which can be “read” by transcription factors such as WT1 (Hashimoto et al., 2016). Moreover, the markedly reduced binding affinity for 5caC and 5mC displayed by WT1 +KTS isoform may demonstrate a splice variant-specific requirement of certain gene promoters to undergo transcriptional activation (Hashimoto et al., 2016). Ergo, this may suggest that oxi-mC presence or absence on promoters and enhancers may perturb or enhance WT1-contingent gene expression, thus contributing WT1-implicated pathologies (Hashimoto et al., 2016). In this context, it is striking that our recent studies have highlighted an enrichment of 5caC not only in certain types of pediatric brain tumors such as medulloblastomas and ependymomas (Ramsawhook et al., 2017) but also in a number of samples of glioblastoma multiforme (Eleftheriou et al., 2015), a cancer previously reported to possess elevated levels of WT1 (Schittenhelm et al., 2008). Considering the observations across the board that 5hmC levels are significantly reduced in malignancies compared to their healthy matched tissue (Figueroa et al., 2010; Ko et al., 2010; Jin et al., 2011), the presence of detectable 5caC in brain tumors may seem rather unexpected and may depend on certain specific features of their methylation/demethylation machinery influenced by overexpression of *WT1* and or *TET2*/*IDH1* & *2* mutations in these cancers.

**Figure 2:**
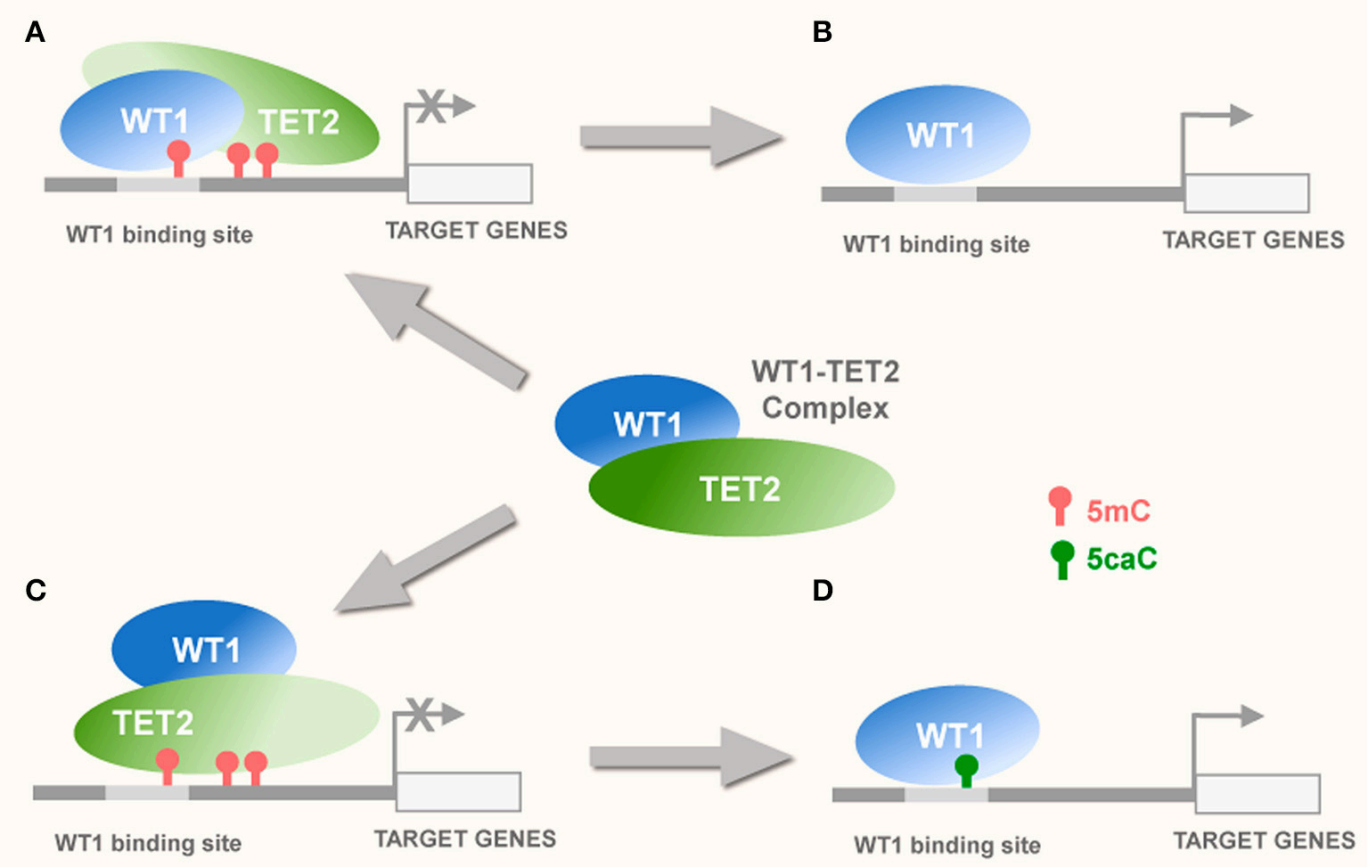
Potential interplay of WT1 with TET proteins**. (A)** WT1 can recruit TET2 to the methylated CpGs within or nearby WT1 binding sites (Rampal et al., 2014) causing demethylation of these CpGs via TET-dependent 5mC oxidation and activation of transcription of the target genes **(B)**. Alternatively, recruitment of TET2 to WT1 binding sequences by WT1 within transcriptionally inactive promoters may lead to oxidation of 5mC to 5caC **(C)**. This may enable WT1 that possesses high affinity for 5caC (Hashimoto et al., 2014), to stabilize this modification and hence potentially facilitate transcriptional activation of its target genes **(D)**.

Collectively, these studies imply two potential modes of WT1 interplay with TET-dependent 5mC oxidation that may influence tumorigenesis. Thus, WT1 can either (1) serve as a binding partner of TET proteins participating in their recruitment to target sequences (Wang et al., 2015) or (2) act as a specific “reader”- of certain oxi-mCs (most notably 5caC) (Hashimoto et al., 2014, 2016; Figure 2). Presently available experimental evidence suggests that both these scenarios may take place during the pathogenesis of brain tumors, particularly glioblastoma multiforme (Schittenhelm et al., 2008; Hashimoto et al., 2009; Eleftheriou et al., 2015; Ramsawhook et al., 2017).

Considering the high mortality rate of this tumor and an immediate need for an efficient strategy for its therapy (Smoll et al., 2013; Thakkar et al., 2014; Ostrom et al., 2015), elucidating potential roles of WT1 and its potential interaction with TETs/oxi-mCs in glioblastomas should represent an important direction for future research. Commensurate with this perspective, two groups in Japan have developed an anti-WT1 peptide vaccine aimed at reducing WT1 protein activity in glioblastoma multiforme patients that is currently undergoing phase II clinical trial (Izumoto et al., 2008; Oji et al., 2016).

Considering the crucial spatiotemporal expression patterns of WT1 as a developmental master-regulator, tumor suppressor and proto-oncogene as well as its potential involvement in the TET/oxi-mCs-related demethylation and transcriptional repression, it is highly likely that ectopic and anachronistic involvement of WT1 in biological processes may perturb normal functioning of cellular machinery, resulting in methylome reprogramming and ultimately, tumorigenesis. Therefore, targeted therapies against WT1 in tumors exiting its over-expression may help curtail disease malignancy and improve patient survival.

## Author contributions

AsR: researched and wrote the initial draft; BC and AR: conceived and edited the final review manuscript. All authors read and approved the final manuscript.

### Conflict of interest statement

The authors declare that the research was conducted in the absence of any commercial or financial relationships that could be construed as a potential conflict of interest.
